# Assessment of the effectiveness of hospital external disaster functional drills on health care receivers’ performance, using standardized patients and mass cards simulation: a pilot study from Saudi Arabia

**DOI:** 10.1186/s12873-024-01095-7

**Published:** 2024-09-27

**Authors:** Nidaa Bajow, Saleh Alesa, Fatima Alzahraa Yassin Shaheen, Abdulaziz Almalki, Ali Alshamrani, Rimaz Alotaibi, Abdulaziz Aloraifi, Carl Montan, Sten Lennquist, Mujahid Alotaibi

**Affiliations:** 1 Disaster Medicine Unit, Emergency Department Security Forces Hospital Program, Riyadh, Saudi Arabia; 2 College of Medicine, Almaarefa University, Riyadh, Saudi Arabia; 3 College of Medicine, Al Faisal University, Riyadh, Saudi Arabia; 4 Department of Vascular Surgery, Department of Molecular Medicine and Surgery, Karolinska Institute, Stockholm, Sweden; 5 International MRMID-Association, Soderkoping, Sweden

**Keywords:** Disaster medicine, Drill, MAC-SIM cards, Realistic scenario, Simulation training, Standardized patient, Team performance

## Abstract

**Background:**

Given the increasing frequency of disasters globally, it is critical that healthcare systems are prepared for these mass casualty events. The Saudi health system’s preparedness for mass casualty incidents needs to be more robust, potentially due to limited disaster drills and inadequate standardized patient (SP) simulation training. This study aims to (i) assess the performance of front-line hospital staff in Saudi Arabia through a functional drill and (ii) evaluate the drill’s effectiveness using SP and MAC-SIM cards, providing detailed insights into its design and execution.

**Methods:**

A functional drill was conducted at a government hospital in Riyadh, Saudi Arabia, on December 19, 2022, using a cross-sectional approach with two phases. 141 healthcare receivers served as subjects, while 23 volunteers acted as SPs. The scenario simulated a building collapse to assess the emergency department (ED) response, interdepartmental communication, and surge capacity. Data were collected through direct observation of healthcare practitioners’ interactions with the SPs, analysis of SP data, and participant feedback. Quantitative data were analyzed descriptively, while qualitative data were examined for patterns and themes related to simulation performance and effectiveness.

**Results:**

The hospital receivers’ performances demonstrated accurate triage categories. The ED team assessed most patients (67%) in less than 5 min. For patients requiring definitive care, such as intensive care unit, 95% spent less than 2.5 h in the ED. Most patients (65%) required ‘other treatments’. Communication was efficient in the triage zone and the yellow treatment area. Participants’ feedback on using MAC-SIM cards during the simulation was overwhelmingly positive with 82.61% reporting that MAC-SIM use helped them respond better. Experienced SPs (paramedics) with prior disaster knowledge and experience outperformed inexperienced SPs (nurses) in the functional exercise.

**Conclusion:**

This groundbreaking study is the first in the Arabic Gulf region to use SPs with MAC-SIM cards in functional drills. The findings highlight the potential of simulation exercises to improve hospital team knowledge and performance when responding to disasters. Multiple evaluation techniques can effectively identify participant strengths and weaknesses, informing future disaster improvement plans. This information is a valuable resource for Arabic and middle-income countries where disaster medicine is still developing.

**Supplementary Information:**

The online version contains supplementary material available at 10.1186/s12873-024-01095-7.

## Background

The frequency of natural disasters has increased worldwide. Between 2000 and 2019, there were 510,837 deaths, and 3.9 billion people affected by 6,681 climate-related disasters alone [1]. In addition to natural disasters such as earthquakes, floods and wildfire, there are also human-caused disasters such as industrial accidents, major traffic incidents and acts of terrorism. Many of these disasters result in mass casualty incidents where large numbers of victims need care. Given the large number of casualties, healthcare systems can often be overwhelmed by the sudden surge in demand for acute and emergency care. As a result, disaster preparedness for these relatively rare but significant events is critical in healthcare systems.

To prevent the occurrence of disaster risk and to reduce existing risk, disaster risk reduction strategies and policies must specify goals and objectives across various timescales with specific targets, indicators, and time frames. National and local plans should include creating or strengthening multi-hazard early warning systems, response capability and personnel, and preparedness plans. Health authorities must strengthen disaster risk prevention and reduction measures and promote the resilience of healthcare facilities to keep the hospitals safe, effective, and operational during and after disasters [2].

Healthcare personnel practice their major incident response skills sparingly, unlike other emergency responders such as police and firefighters. As a result, their skills in these areas may more closely resemble those reported by volunteers than professional responders [3]. The core concept within new disaster medicine education is simulation-based training, which has outstanding potential for producing effective learning outcomes [4]. Exercises are believed to assist in identifying concerns with the health system and clarifying healthcare personnel’s roles and responsibilities [5–7]. The primary benefit of participating in drills is the opportunity to rehearse using a major incident plan. Since mass casualties are rare but highly consequential crisis scenarios, simulation exercises are the only way to test major incident plans, maintain current response knowledge and skills, build systemic response capabilities [8], and prepare healthcare workers for novel circumstances [9, 10]. Two meta-analyses have found that simulation exercises improve knowledge [11]. However, more research is needed to advance the understanding of this field, evaluate healthcare professionals’ readiness, and determine the hospital readiness levels for various crisis scenarios. Regardless of the type of intervention, when these drills accurately simulate real-world occurrences, they have been found to improve hospital disaster preparedness [12–15].

The MAC-SIM (Mass Casualty Simulation Cards) system was developed for interactive training in the international MRMI (medical response to major incidents) course, covering the whole rescue chain. It has been used for training more than 5000 responders of different categories worldwide in the last ten years and has been thoroughly evaluated and validated concerning its accuracy [16]. While this system has proven beneficial for disaster preparedness training, a systemic review done in 2020 showed no research conducted to investigate the use of standardized patients with simulation exercises in developing countries despite the high frequency of disaster occurrences in these countries and the ensuing requirement that medical professionals participating in emergency responses be adequately prepared to handle large numbers of casualties [17]. The use of standardized patients with mass card simulation was only noted in Sweden [18, 19].

Using simulated standardized patients (SPs) in full-scale exercises is crucial because it can have realistic effects on participants. SPs refer to actors who play specialized roles as patients or patients with certain conditions to simulate a real-world scenario [17]. SPs were first used in medical education in 1964 [20] to evaluate physicians’ professionalism. SPs are commonly used in symptom simulation techniques to ensure an accurate replication of reality. Information is gathered either through the actor’s active response (verbal and motor) or using a card hung around the SP’s neck containing signs, symptoms, vital signs, and the patient’s history [21–23]. Despite being used for many years in medical education, the effectiveness of SPs in exercises has yet to be fully clarified.

Saudi Arabia is a developing nation that places the nation’s health at the center of emergency and disaster risk management. In March 2015, Saudi Arabia, along with 22 Arab states, adopted the Sendai Framework for Disaster Risk Reduction (SFDRR) 2015–2030. On 15 April 2018, Saudi Arabia adopted the Arab Strategy for Disaster Risk Reduction (ASDRR) 2030. Despite adopting the SFDRR and ASDRR, in 2022 many hospitals in Saudi Arabia showed deficits in disaster management, which were evident due to limited disaster drills and insufficient training using SP simulations, particularly in hospital staff education. Most hospitals still need to hold awareness sessions or disaster health workshops [24, 25]. Saudi hospitals also require disaster drills in their emergency preparedness plans or exercises involving coordination and communication with other disaster organizations [26–29]. In short, the Saudi health system demonstrates that “Preparedness without proper education and training is not preparedness“ [30].

While specific epidemiological data on the use of SPs in disaster simulation drills for Saudi Arabia is limited, the global burden of disasters, coupled with the increasing frequency and severity of such events, underscores the importance of this study. Developing countries, including Saudi Arabia, are particularly vulnerable to disasters, and their healthcare systems often face significant challenges in managing mass casualty incidents [25]. The effectiveness of standardized patient simulations combined with the MAC-SIM system in enhancing the performance of Saudi hospital staff during disaster response drills is crucial because it addresses a critical disaster preparedness gap and can significantly improve patient outcomes and the Saudi healthcare system resilience.

Theoretical frameworks in disaster risk management emphasize the importance of simulation-based training and SPs as practical tools for enhancing preparedness [17, 24, 25]. The lack of research on this topic in the Saudi context highlights the need for this study to fill the knowledge gap and inform the development of improved disaster response strategies.

This study aims to evaluate (i) the performance of Saudi front-line hospital healthcare receivers (hospital staff) through a functional drill of the hospital’s disaster response and (ii) the effectiveness of the functional drill using SPs and MAC-SIM cards. By providing detailed descriptions of the drill’s construction and execution as well as the data collection process, this study also provides evidence-based instructions and recommendations for enhancing disaster preparedness in Saudi hospitals through the effective use of SP simulations and the MAC-SIM system. The findings can inform the development of training programs, protocols, and guidelines for improving hospital disaster response capabilities nationwide.

## Methods

This groundbreaking study used a cross-sectional intervention approach, which consisted of two fundamental phases: preparation and implementation.

### Setting

The primary response-related departments of the Security Forces Hospital program in Riyadh [Emergency department (ED), Wards, Intensive Care Unit (ICU), Operating room (OR)].

The Security Forces Hospital (SFH) - Riyadh is a government tertiary hospital with approximately 550 beds and over 250,000 patients who visit the ED annually. Twenty-three students volunteered to act as standardized patients (SPs). The 2021 updated hospital disaster plan was in use at the time of the study. Standard operating procedures with a surge capacity plan were evaluated in the primary response-related departments, whereas the physical movement of SPs occurred exclusively in the ED.

### Participants

- **Players** (trainees): **141** front-line hospital receivers participated in the drill; the ED staff actively participated in the disaster drill, with **47** individuals involved. This group included 22 nurses, 12 physicians, three paramedics, five administrators (covering patient eligibility), and five security guards. The drill coordinator requested the heads of response-related departments, such as OR, ICU, and wards, to assign on-duty staff (**89** personnel) to test the surge capacity plan for each department. This plan evaluates four elements: staff, stuff, structure, and systems. **Five** individuals from different medical and administrative departments activated the hospital incident operation room. The sample of players involved in the training was collected randomly using probability sampling, specifically stratified random sampling.
- **Instructors**, **controllers**, **and evaluators**: **Five** instructors and **controllers** from the drill control team were responsible for the scenario injects (or master event list), actors’ (SPs) actions, and players’ organization. **Six** EMS student volunteers assisted the control team in their tasks. **Fifteen** evaluators contributed to the drill; five were invited from three different government sectors (King Saud University, King Abdul-Aziz Medical City, and King Fahad Medical City), all with previous experience in emergency and disaster medicine education.
- **Actors**: **23** volunteers (13 paramedics and 10 nurses) acted as standardized patients (SPs) in this drill. Each SP’s occupation and prior experience were recorded. Before conducting the drill, the ED Director sent invitation letters to Prince Sultan College-Riyadh (medical services) and the hospital office (nursing internship training) to select volunteers to act as SPs. All SPs (actors) consented to participate before the functional drill. Attendance was compulsory for all actors once recruited.

The study was approved by the institutional research board of King Abdullah bin Abdulaziz University Hospital at Princess Nourah bint Abdulrahman University (IRB. Log. NO 23-0173E).

### Drill development

#### Phase 1: the preparatory phase

This phase was established by six members of the disaster subcommittee with experience in emergency and disaster medicine. The experts set the drill’s goals and parameters according to the latest hospital risk assessment report, hospital resources, and the backgrounds and skills necessary for performing the roles and activities specified in the drill.

The first author developed the following three stages for Phase 1:

**Stage one for Phase 1** is about verifying the requirements to set up a drill or the conditions needed for conducting exercises. Before preparing training, one needs to be sure that specific criteria are met, such as the following [31–33]:

- Availability of an emergency management organizational structure with a disaster response plan (**Hospital Incident Command Structure**).
- The items that must be investigated during the drill.
- A risk scenario that considers potential dangers, weaknesses, and resources.
- A site where there is little risk to participants and where the physical and environmental circumstances are appropriate for simulating emergencies and.
- Necessary logistical support and institutional financial resources.

##### Stage two for phase 1

**Fig. 1:**
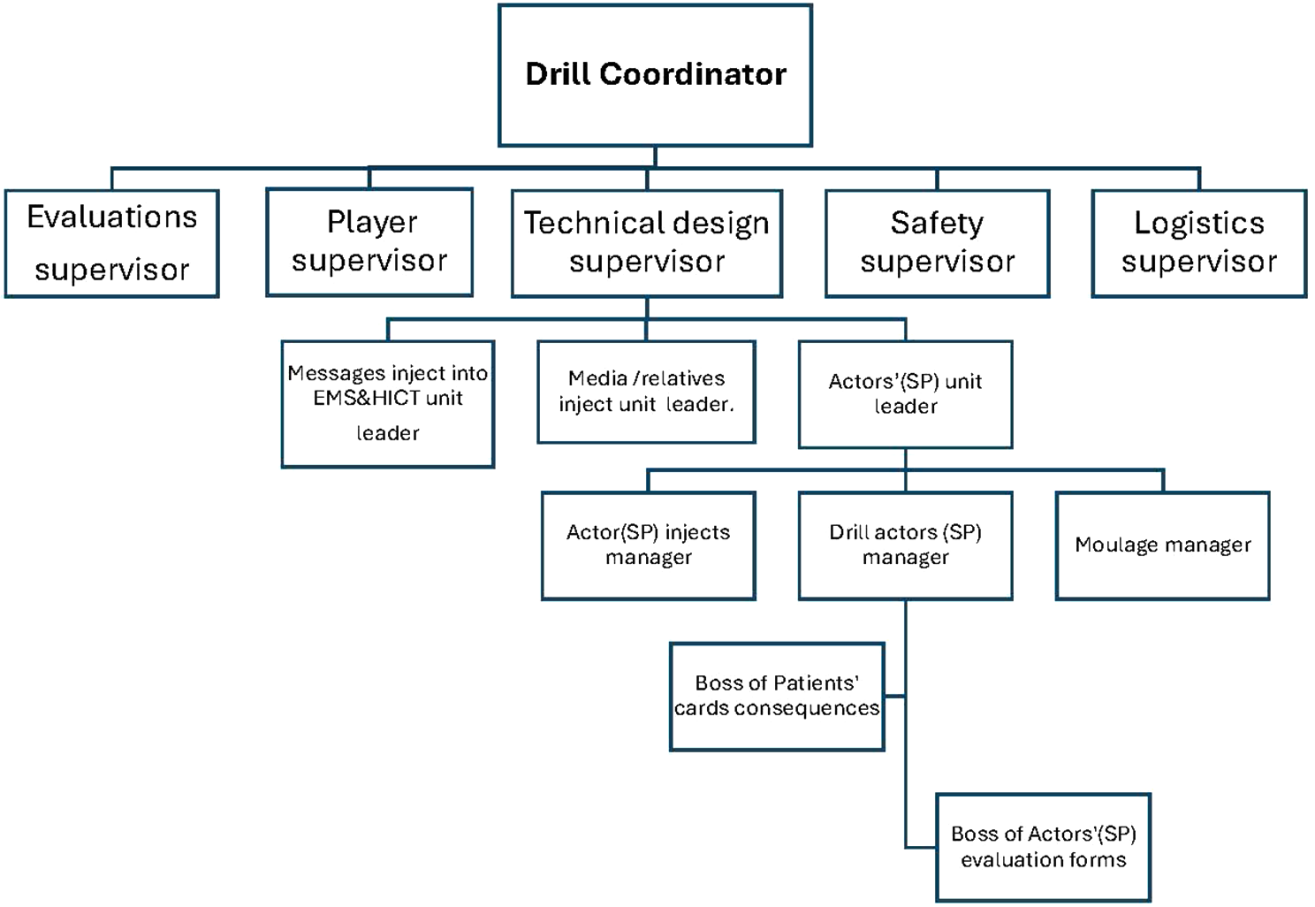
Organizational structure. *EMS*: Emergency Medical Services, *HICT*: Hospital Incident Command Team

The organization process integrates and coordinates the work of different teams in developing the drill, which is conducted by the drill coordinator and includes a specific structure (Fig. 1 shows the organization structure).

In Fig. 1, each position has tasks of responsibility, including technical and logistical aspects of the emergency drill. The technical design section is responsible for the master event list that explains the inject with action time, event input, action required by responsible parties/departments, duration for each inject/event during the scenario, and resource requirements.

Situation reports with timelines were also prepared for the media, relatives, and the local emergency operation center. Finally, the technical team wrote the scenario according to the abovementioned processes [31, 32].

**Stage three of Phase 1** (preparedness) involved preparing evaluation instruments, training SPs, and creating the simulation cards.

A. Instrument preparation

The technical design team prepared the evaluation forms under the supervision of the drill coordinator. Five different forms were used to evaluate the functional drill’s primary goals.

The first form involved collecting general feedback from the player, facilitator, and evaluator on the drill. The form evaluates the following items: the organization of the drill, the realism of the scenario itself, the orientation lectures before the functional drill, the level of facilitators, the hospital’s interdisciplinary approach, and the effect of utilizing standardized patients with MAC-SIM cards.

The second form evaluated the ED team’s performance in the triage zone and treatment (red, yellow, and green) areas, the security team’s response, communication flow, and the emergency operation center with the HICT (hospital incident command team) by external and internal evaluators.

The third form involved evaluating the performance of the ED team using actors (SPs) in the ED areas. It specifically focused on the accuracy and efficiency of the triage team’s triage, the time patients spent waiting for examination by the ED team, and the time it took to transfer patients from the ED. It also included an open-ended question about how to improve the drill design.

Instructors also assessed the actors’ performance (fourth form) to determine if they achieved the scenario’s objectives, understood their roles and behaviors, and adapted their physiological parameters to the players response management.

The last form pertained to the surge capacity protocol plan for response-related departments such as the ED, ICU, OR, and wards.

The first form was modified from the Public Health Emergency Exercise Toolkit [30]; the second combines the Ministry of Health (MOH) and the County of Los Angeles Department of Health Services Emergency Medical Disaster Services drills forms [31]. Three experts in disaster medicine education prepared for this study and validated the third and fourth forms. Ten paramedic students not included in the SP team participated in the pilot study for the third survey. The last questionnaire was developed around a questionnaire piloted and published by the medical response to major incidents and disaster course [16, 18].

An ED key treatments list was created for patients, primarily focusing on (A) airway, (B) breathing, (C) circulation, and (O) other treatments, such as immobilizing fractures and dressing wounds. The list indicated the start time for completing each required treatment. It was placed at each bedside so that healthcare providers could document their patients’ main treatment, including examination and planning for surgery and therapy.

B. Standardized patient training and moulage trials

Two simulation techniques were used: standardized patients (SPs) and MAC-SIM to identify victim symptoms and signs.

Individuals with previous training in disaster medicine or drill experience were known as **experienced SPs**. The term “**inexperienced SP**” refers to SPs who have never been in a drill or received training in disaster medicine.

The drill design team delivered an orientation session before the drill, explaining scenario objectives, details with a master event list, and storyboards with MAC-SIMs for each SP. At the following meeting, the disaster medicine expert instructed the actors (SPs) on simulation techniques (such as acting and assessing participant reactions). The storyboards described the outcome of the patient’s situation and whether he received treatment or not. Ten days before the drill, two moulage experts applied the makeup descriptions to the makeup collector cards for each SP. Moulage experts from the medical field were invited to vividly describe wounds or illnesses on SPs to create highly realistic scenarios.

C. Mass card simulation

The MAC-SIM is a validated system developed for assessing and comparing various triage methods in major event responses [18]. The actors (SPs) wear simulation cards around their necks to represent their signs, symptoms, and physiological parameters in line with their reactions to the healthcare provider’s medical response.

The physiological parameters (Airway, Breathing, Circulation, and Disability) used by Advanced Trauma Life Support are employed to depict the patient’s condition along the edges of the card [18].

**Fig. 2:**
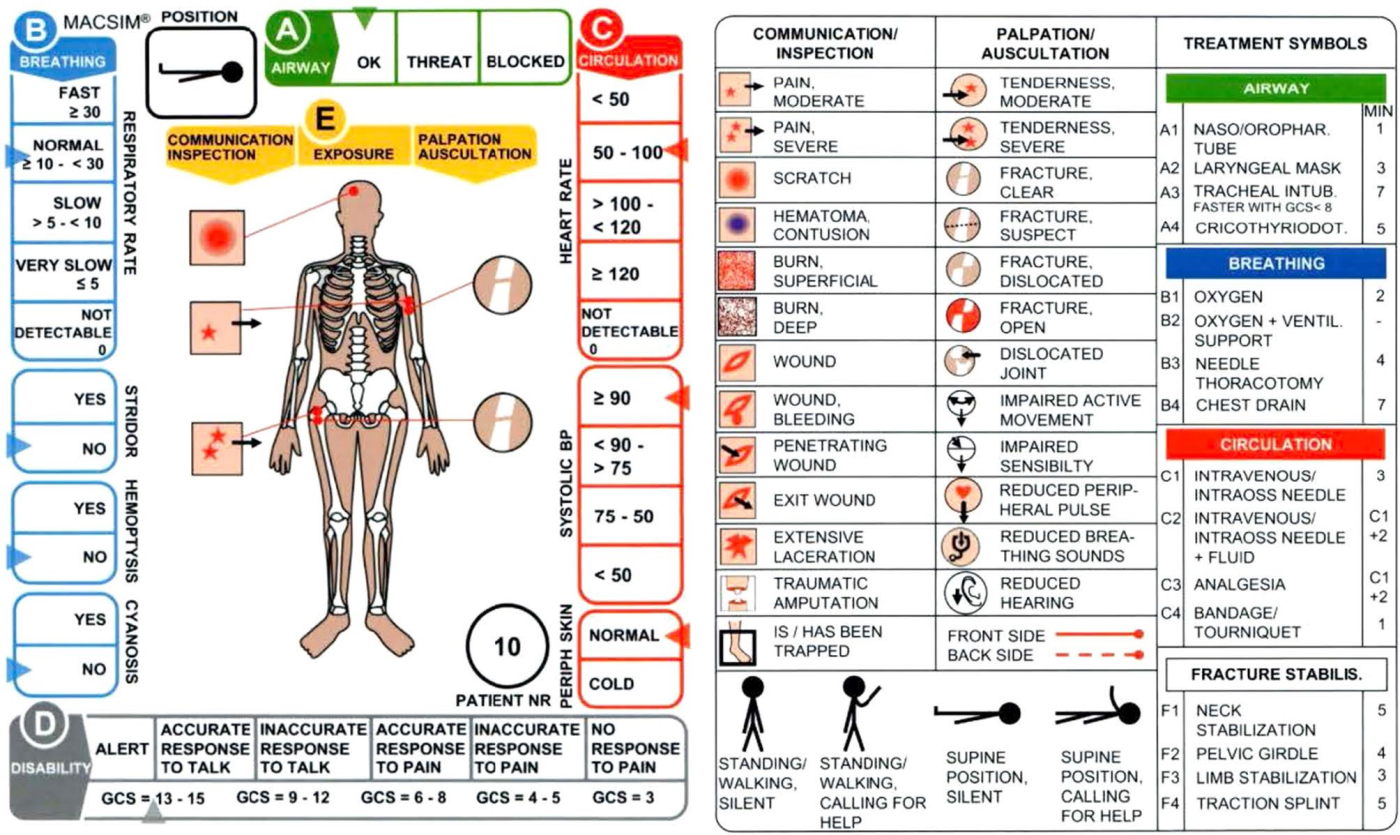
A Mass Casualty Simulation card (MAC-SIM)

Instructors can adjust the criteria based on the time since the injury and whether any therapies were provided. The card includes a transparent symbol system at the center (Fig. 2), making it easy to represent the various injuries combined with SP moulage. Additionally, the card includes the patient’s starting position, age, and gender. Two sizes of cards are available: a larger one for attaching to casualty actors during field exercises and a smaller one for simulation use. The technical leader provided the instructor with information about each patient:

- The entire and definite diagnosis of all injuries.
- The proper time for various maneuvers to be performed to reduce the risk of complications and fatality.
- Results in the best-case scenario, i.e., if the patient had recovered fully and all appropriate treatments had been administered.
- Possibility of need for ventilator treatment.
- Trauma scores to correlate results with Injury Severity Score (ISS).
- Revised Trauma Score (RTS) [16, 18, 19].

Storyboards with cards were prepared for 23 cases: five reds, 12 yellows, and six greens. The green cases were deemed self-evacuation casualties by private cars to the ED entrance.

### Phase 2: the implementation phase

#### Stage one: orientation session

The drill coordinator team held a one-hour orientation lecture ten days before the drill. The lecture covered the objectives of the drill, organizational structures, and critical information from the disaster manual. The presentation was delivered to response-related departments, focusing on the ED team. The presentation also included essential information that needed to be understood before the drill, such as an ED floor map, the ED surge capacity plan, ward discharge criteria, and safety plans in the event of an incident. The instructors introduced a tool for treating disaster victims and the primary (start) and secondary triage procedures. Finally, the hot wash and after-action reports procedures were explained.

#### Stage two: conducting the drill

On December 19, 2022, at 11 a.m., a functional drill was conducted inside the hospital. The scenario involved a building collapsing during a graduation ceremony, resulting in over 60 casualties. The renal dialysis area was utilized as the incident scene.

When the initial report of the disaster was made from the regional command center, it took 17 min to be clarified in which time the ED standby level was activated. Six self-evacuated victims left the incident scene by private vehicle during the standby period. After receiving confirmation that 17 urgent and immediate cases had been transported to the hospital, the ED director-initiated level two of the disaster plan. Each patient evacuated by ambulance to the hospital’s ED was transferred from the renal dialysis area to the ED. Ten evaluators were assigned to different areas, such as the command control room, triage, and treatment areas. Four of these evaluators checked the physiological parameters of each victim to ensure they were consistent with the treatment or life-saving procedures received during the drill. Additionally, five head nurses were allocated to departments such as the ICU, wards, and OR to assess the surge capacity plan in each department. After the last patient arrived at the ED, the coordinating chief announced that the external disaster drill was over with the remainder of the drill focused on the internal responses. The entire simulation lasted two and a half hours and focused on the ED response and internal communication within and between hospital departments.

Thirty minutes after the drill ended, the drill coordinator, players, and the evaluation team participated in a one-hour hotwash session and completed the forms for the drill feedback. Standardized Patients (SPs) also joined instructors for a brief discussion to address the positive and negative aspects. One week after the drill, the after-action report was sent to the medical administration and disaster committee.

### Analysis Method

Data were analyzed using IBM SPSS version 25 (Statistical Package for the Social Sciences). Two types of analyses were employed: reliability tests and non-parametric Kruskal-Wallis tests. The reliability test involves using Cronbach’s alpha to measure the consistency of a questionnaire, mainly through Likert-scale questions.

## Results

### Demographics

The actors (SPs) consisted of 13 male paramedics with a history of attending awareness courses, including disaster drills, during their college education (experienced SPs) and ten nurses. The ten nurses had yet to participate in disaster courses or exercises (inexperienced SPs). The source population comprised 141 healthcare workers and players (trainees) from the Security Forces Hospital in Riyadh. These healthcare workers included medical, nursing, and paramedical staff working in the hospital.

### Reliability of data

The reliability test, Cronbach’s alpha coefficient, is a tool used to measure the internal consistency of Likert-scale questions. It examines the reliability of all the statements in scaled questions. The reliability of the drill results yielded a Cronbach’s alpha coefficient value of approximately 0.887. This indicates that the study’s data are reliable.

### Evaluation of players (medical staff) by the actors (SPs)

**Fig. 3:**
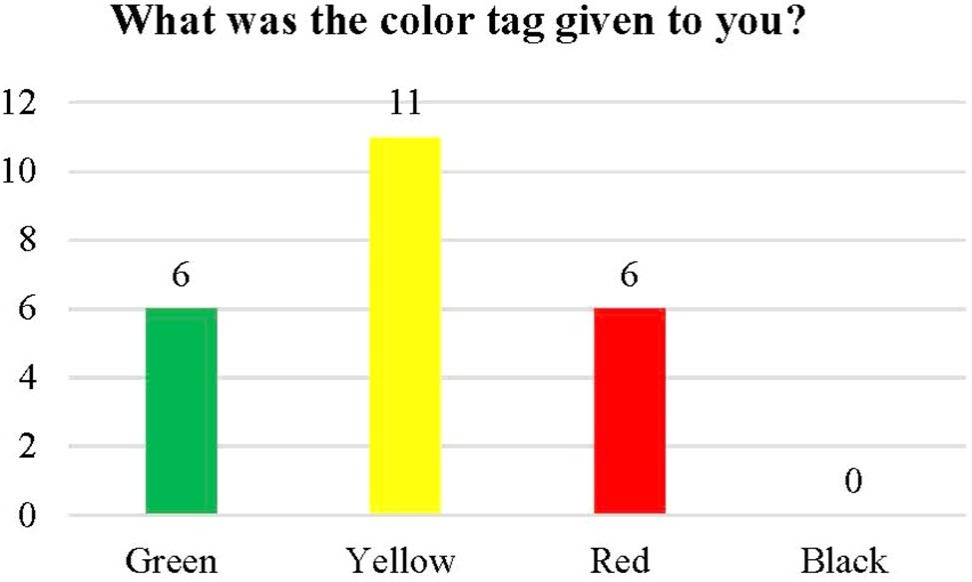
Color tags given to standardized patients (SPs)

The feedback from the actors (SPs) concerning their categorization by players (medical staff trainees) in the hospital triage team at the triage station and the color tag assigned to each SP is displayed in Fig. 3. The triage category for each SP was correct. At first, there were five red cases, but a sixth case appeared after a myocardium infraction in one of the yellow cases, which turned red in the yellow treatment zone due to a delay in treatment.

**Fig. 4:**
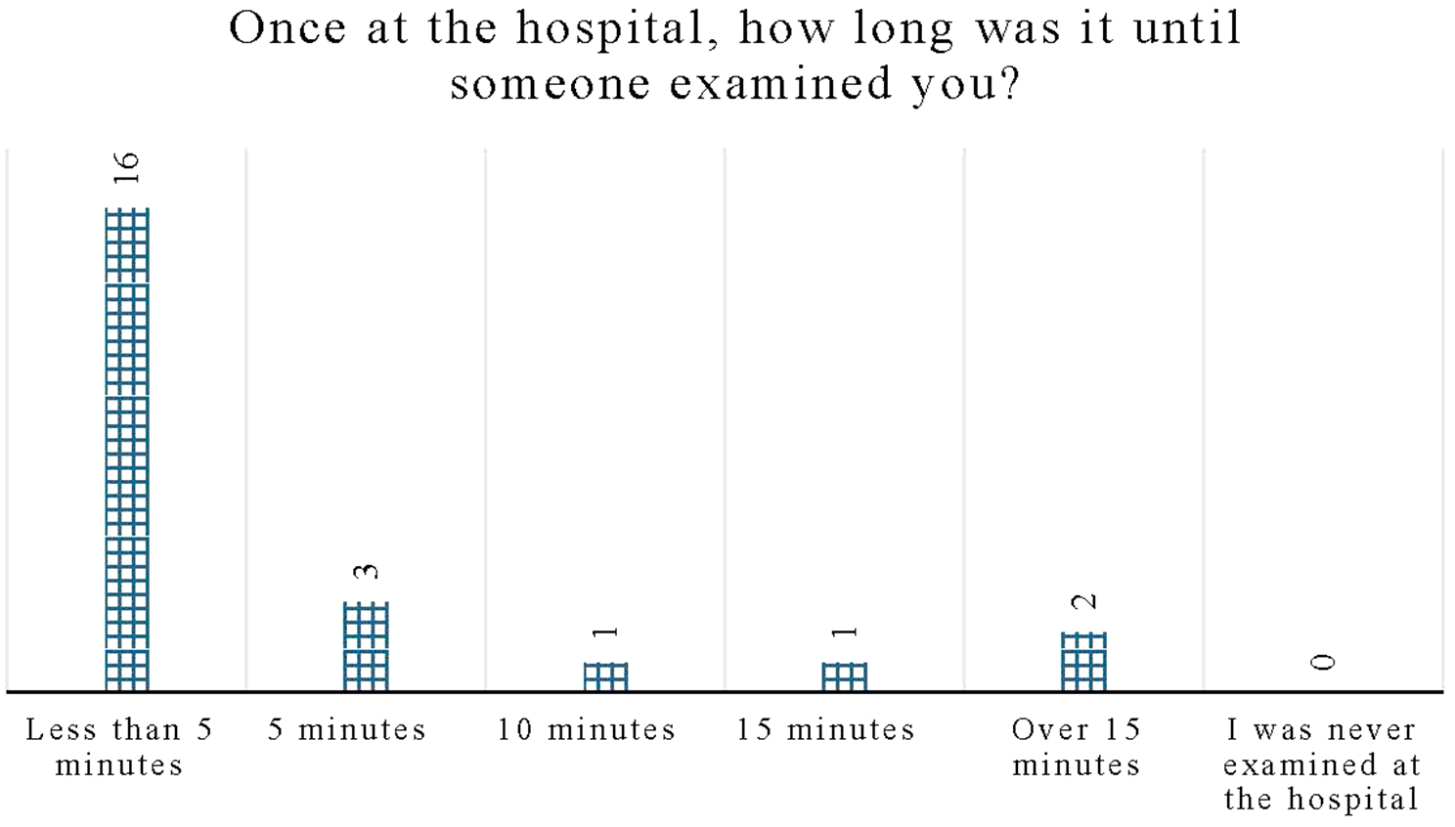
Time taken to examine standardized patients (SPs) once in the hospital

The time taken for the ED team to examine each patient showed the majority, 67% (16), were seen in less than five minutes, 13% (3) in 5 min, and 9% (2) in more than 15 min. Figure 4**illustrates** the time it took for a participant to examine each SP once they arrived at the hospital.

**Fig. 5:**
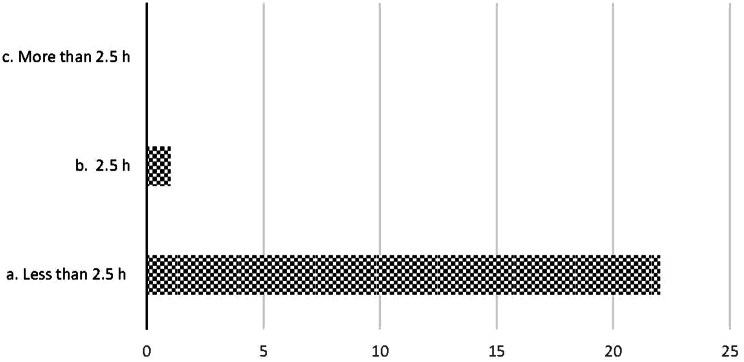
Time spent in the ED before the standardized patient (SP) was sent to OR /ICU /Ward

For the SPs destined for definitive care, including ICU, OR, and wards, 95% spent less than 2.5 h in the ED before they were sent to definitive care, as shown in Fig. 5.

Assessment of the hospital disaster plan implementation by all involved parties.

### I. Communication and information flow in the triage and treatment areas

**Fig. 6:**
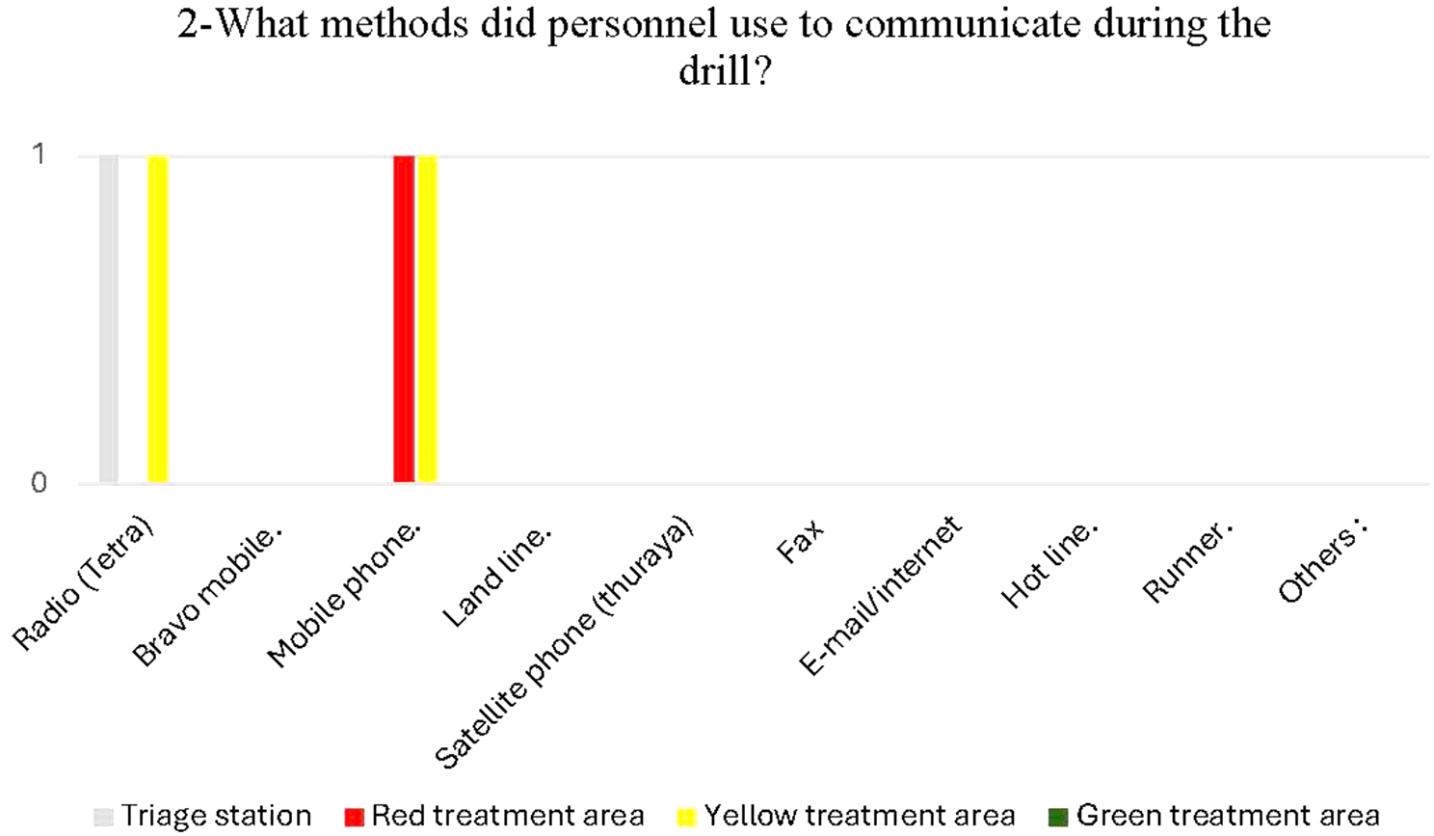
Methods personnel used to communicate in different areas during the drill

The evaluators assessed the communication effectiveness and found it efficient in the triage and yellow treatment areas but not in the red and green areas. Radio (Tetra) was the most popular form of communication in the triage zone and areas receiving yellow patients, as shown in Fig. 6.

Hospital mobile phones were used in yellow and red areas. None of the hospital communication tools were used in the green zone, as the staff primarily relied on their personal mobile phones, which did not receive messages from the hospital. Additionally, an unexpected battery stoppage resulted in the inability to use the radio in the green and red areas.

There were communication breakdowns in various hospital areas, but the severity varied. The issues in the triage zone were mainly between team members and treatment area coordinators, while those in the red area were primarily with other departments. There needs to be more communication between different departments and other treatment area coordinators in the yellow area. More information flow was required for the green area, and essential information was only partially obtained in all areas, including the triage zone. Updates on the situation were only sent to the triage area, such as the number of victims arriving and the status of the disaster occurrences, through calls from the hospital incident commander. The red and yellow treatment areas were also only partially informed of the current general condition inside the hospital.

### II. Procedures implementation

After the level two disaster was activated, the team leader took charge of the red zone in 7 min and the leader of the yellow zone in 12 min. However, the hospital staff needed to be prepared for all three treatment areas: red, yellow, and green. The hospital disaster plan was only partially applied in the red and yellow regions because some players (medical staff) needed to check their action cards to understand their roles and responsibilities.

### III. Medical treatment interventions

The types of medical interventions most frequently needed by patients in the ED treatment areas were investigated statistically using the non-parametric Kruskal-Wallis test as the data were not normally distributed (Shapiro-Wilk test statistic = 0.722, *p* = .001). The results showed that there was a statistically significant difference between the groups, with most patients requiring “other additional treatments,” such as fracture immobilization, pain, and anxiety management (65%), followed by breathing interventions (13%), circulation interventions (13%), and airway interventions (8.6%) (Table 1).

**Table 1:** Required treatment for patients

Required maneuvers	Count	Percentage
Patient required airway (A)	2	8.6%
Patient required breathing (B)	3	13%
Patient required circulation (C)	3	13% one MI BECAME RED
Other (O), e.g., fracture immobilization, pain management	15	65%

### IV. Surge capacity plan activation

The ED director activated the standby level within 5 min. Seventeen minutes after the first notification, level two was activated, and most hospital incident command team members were available in the hospital emergency operation center. The critical capacity information, including for the OR and ICU, was sent to the regional command center 3 min after level two activation. The flow of communication with the regional command center and intradepartmental was excellent.

The ED was the only department that communicated and interacted with SPs. According to level two of the plan, an additional team from another response-related department should be moved immediately when they receive notification and assigned to areas with duties consistent with their skills. However, only five physicians and seven nurses from other departments joined the ED team in the red and yellow areas one hour after activation of the hospital disaster plan. The main issue affecting the arrival of additional staff to the ED was communication failure because SMS messages are sent only to the hospital’s mobile system, not personal mobiles.

The treatment list showed overused resources; some procedures were unnecessary, and some, such as chest X-rays, could be done in the wards to help alleviate surge in the ED.

The first patient was sent to the ICU 40 min after the activation of the emergency level. At that time, three ventilators were available. The ICU team reviewed and selected four patients for stepdown to alternative wards. The number of available staff (nurses, respiratory technicians, and doctors) was fair compared to the number of occupied beds (18 out of 21). For the OR, the head nurse immediately asked to evacuate post-operation-ordinary patients and transfer the area to the preoperational zone. Two ORs were available for use by disaster cases at that time; there was no lack of supply and staff.

The head nurse and the medical team in the surgical ward identified 21 patients during the drill for discharge to their homes based on reverse triage.

There were 99 unstaffed but licensed beds and six rooms with sofas that could be used for disaster patients. The best organization and coordination were observed in the control room and ICU, followed by the ED. Staff abstention and unawareness of their roles and responsibilities were observed mainly in the wards. The redistribution of patients to different units on alert was good.

## Effectiveness of the functional exercise using SPs and MAC-SIM cards

### I. Assessment of SPs after the drill

According to Pimentel (2010) [34] the five-point Likert scale is an interval scale. A mean of 1 to 1.8, means strongly disagree, from 1.81 to 2.60 means disagree, 2.61 to 3.40 means neutral, from 3.41 to 4.20 means agree and from 4.21 to 5 means strongly agree. As the data were not normally distributed, a Kruskal-Wallis test was utilized to compare the responses from the different groups (Table 2). The descriptive statistics for the summary results of the instructor assessment of SPs after the drill are shown in Table 3.

**Table 2:** Comparison of responses from different groups regarding their understanding of the aims and objectives of the drill (Q1)

Group (Knowledge Level)	Kruskal-Wallis H	df	Asymp. Sig.
2-Actors(SP) know Role and behavior	13.123	2	0.001
3-Actors (SP) know Action of simulators	12.351	2	0.002
4-Actors (SP) know Change the physiological parameter according to response of players?	9.541	2	0.008

**Table 3:** Summary results of instructor assessment of SPs after the drill

Statements	*N*	Minimum	Maximum	Mean	Std. Deviation
1- (SP) know Aim and objectives of exercise	23	3	5	4.09	0.42
2- (SP) know Role and behavior	23	3	5	4.00	0.43
3- (SP) know Action of simulators	23	3	5	3.91	0.60
4- (SP) know how to Change the physiological parameter according to the players’ response?	23	2	5	3.30	0.82

*Note* 5 strongly agree, 4 agree, 3 neutrals, 2 disagree, 1 strongly disagree

Most instructors agreed that the SPs understood the aim and objectives of the drill (mean 4.09 +/- 0.42) and understood their roles and behavior (mean 4.00 +/-0.43). Most instructors were also satisfied with SP performance (mean 3.91+/-0.60). Most instructors reported neutral evaluations of whether SPs knew how to change their physiological parameters according to the players’ (medical staff) responses (mean of 3.3+/- 0.82).

### II. Comparison of the performance of experienced and inexperienced SPs

There was a difference in the performance of experienced and inexperienced SPs during the drill exercise (Table 4). The results indicate that paramedic SPs with previous disaster knowledge (previously attended disaster awareness sessions and participated in disaster exercises at university) generally performed better in the functional drill than nurse SPs.

**Table 4:** Comparison between experienced (paramedics) and inexperienced (nurses) performance as standardized patients (SPs)

Actors’ assessment questions	Work type	*N*	Mean	Std. Deviation	F	Sig.
1-(SP) know Aim and Objectives of exercise	Nurse	10	4.0000	0.00000		
	Paramedic	13	4.1538	0.55470	10.589	0.004
2- (SP) know Role and Behavior	Nurse	10	4.0000	0.00000		
	Paramedic	13	4.0000	0.57735	4.058	0.057
3- (SP) know Action of Simulators	Nurse	10	3.9000	0.31623		
	Paramedic	13	3.9231	0.75955	5.409	0.030
4- (SP) know ow to change the physiological parameter according to response of players?	Nurse	10	3.0000	0.00000		
	Paramedic	13	3.5385	1.05003	27.916	0.000

While paramedics performed slightly better in understanding goals and adjusting simulator parameters, these differences were not statistically significant. Both paramedics and nurses had a good grasp of the simulation scenario and their roles within it. However, paramedics were more able to adapt the simulation based on player (medical team) responses, suggesting they might be more adept at handling dynamic situations in real-world emergencies.

### III. Using MAC-SIM cards and SPs during the drill

Participants (players, evaluators, and controllers) feedback regarding the use of MAC-SIM cards during drill simulations was generally positive, with over 86% of respondents finding them helpful. The remaining participants found them partially beneficial. Similarly, approximately 82% of respondents believed using SPs during the drill simulation helped them respond better, while the remaining respondents said it only helped slightly.

### A. General drill feedback

Only 23 (16% of drill participants) answered the overall post drill survey (Table 5). Defining agreement or strong agreement with the given statement as a positive evaluation, 100% of the respondents found that the drill was appropriate for their role, 87% reported that the drill scenario was realistic, and 82.6% found that the controller(s) were knowledgeable about the material and kept the drill on target. The same proportion (82.6%) found that the level and mix of disciplines and participants included the right people for this exercise.

**Table 5:** Drill feedback assessment by all participants (1 = strongly do not agree, 5 = strongly agree)

Assessment	1	2	3	4	5
1- The drill was well organized and structured.	0.0%	0.0%	30.4%	26.1%	43.5%
2- The drill scenario was realistic.	0.0%	0.0%	13.0%	17.4%	69.6%
3- The briefing and/or presentation helped me understand and become engaged in the scenario.	0.0%	17.4%	26.1%	17.4%	39.1%
4- The controller(s) was knowledgeable about the material and kept the drill on target.	0.0%	0.0%	17.4%	30.4%	52.2%
5- Participation in the drill was appropriate for my role.	0.0%	0.0%	0.0%	34.8%	65.2%
6- The level and mix of disciplines and participants included the right people for this exercise.	0.0%	0.0%	17.4%	21.7%	60.9%

In the open-ended question about the issues and needs for improvement, most of the comments focused on communication, i.e., equipment failure (unreceived SMS messages, radio unable to work in some areas due to battery stoppage), the flow of information, including briefing and debriefing sessions, and the need for workshops, training, and awareness lectures before the drills launched. There was also a request to design a small job action card to hang beside the identification cards to summarize and remind the team of the main tasks during a disaster response.

#### i. Comparison of the responses from the different participant groups

Table 6 presents the response answers of each participant group (players, evaluators, and controllers) regarding the drills. The players group reported the highest mean score for the realism of the scenario (4.625 ± 0.719) compared to the evaluators and controllers (4.400 ± 0.894) (4.500 ± 0.707). The mean score for participation in the drill was also highest in the player group (4.688 ± 0.479) compared to the mean scores of other respondents (evaluators and controllers).

**Table 6:** Comparison of the drill feedback results between different groups

Report													
Role		1- The drill was well organized and structured.		2- The drill scenario was realistic.		3- The briefing and/or presentation helped me understand and become engaged in the scenario.		4- The facilitator(s)/controller(s) was knowledgeable about the material and kept the drill on target.		5- Participation in the drill was appropriate for my role.		6- The level and mix of disciplines and participants included the right people for this exercise.	
Player	Mean		4.1250		4.6250		3.7500		4.2500		4.6875		4.5000
	N		16		16		16		16		16		16
	Std. Deviation		0.95743		0.71880		1.23828		0.85635		0.47871		0.73030
Evaluator	Mean		4.0000		4.4000		3.8000		4.6000		4.6000		4.4000
	N		5		5		5		5		5		5
	Std. Deviation		0.70711		0.89443		1.09545		0.54772		0.54772		0.89443
Controller	Mean		4.5000		4.5000		4.0000		4.5000		4.5000		4.0000
	N		2		2		2		2		2		2
	Std. Deviation		0.70711		0.70711		1.41421		0.70711		0.70711		1.41421
Total	Mean		4.1304		4.5652		3.7826		4.3478		4.6522		4.4348
	N		23		23		23		23		23		23
	Std. Deviation		0.86887		0.72777		1.16605		0.77511		0.48698		0.78775

As the data were not normally distributed (Kolmogorov-Smirnov test *p* < .001), Kruskall-Wallis tests were used to compare the responses of the different groups. There was a significant difference in the mean drill response from all participants without the SPs (*p* < .001). The findings of the participant input in the drill showed that statement 3 (The briefing and presentation made it simpler for me to understand and participate in the situation) has the highest standing.

#### ii. Standardized patient (SP) feedback

None of the SPs reported observing any problems during their participation in the drill or any improvements needed; most answered that there were no issues in the drill design or aspects needing improvement.

## Discussion

This cross-sectional pilot study evaluated the performance of healthcare receivers and the effectiveness of functional drills using standardized patients (SPs) and MAC-SIM cards in a major government Saudi Hospital. The World Health Organization highlights the significance of hospital readiness and response capability [35–37], considering hospitals’ role as essential parts of the healthcare system in responding to emergencies and disasters. The maintenance of service continuity and effective delivery of hospital preparedness plans and crisis management techniques can reduce death rates significantly [38].

The Central Board of Accreditation for Healthcare Institutions in Saudi Arabia now requires mandatory disaster preparedness and training [39]. This functional drill was conducted three years after the COVID-19 pandemic and required extensive readiness, including multiple evaluation techniques, to assess the team’s performance. The evaluation process for disaster medicine education and training varies due to the diverse nature of disaster patterns and the factors that affect the response. This diversity results in the need for different evaluation techniques and forms compared to other medical disciplines [16, 40]. Therefore, various methods were utilized to evaluate the effectiveness of the disaster medical training programs, as not all approaches can sufficiently measure their usefulness, as demonstrated by previous research [41–44].

### Drill evaluation approaches

In the current study, the drill preparation team considered the strengths and weaknesses of each evaluation approach and its alignment with the goals of the functional drill [44]. Previous studies have emphasized that using a single evaluation technique is insufficient for a comprehensive assessment of an activity; instead, it is preferable to use a combination of evaluation methods to analyze multiple aspects of an index or performance [44, 45]. The five evaluation methods were based on quantitative and qualitative data to provide valuable insights [46]. The resulting data were reliable (Cronbach’s alpha value = 0.887); however, the disaster drill evaluation method could be improved by considering several factors, such as its function-based nature, accuracy, transparency, reliability, validity, and ease of use [41, 43, 47].

This study evaluated the hospital drill and the team’s performance from various perspectives. The evaluation was initiated by gathering participant feedback about the drill organization, scenario realism, instructor effectiveness, and orientation sessions. This feedback is crucial for improving future drills. Other evaluations focused on medical treatment, including triage accuracy, time for patient assessment, and time spent in the emergency department before transfer. The essential aspects of the disaster manual, such as communication, organization, and surge capacity plan, were also observed. In another study, the drill team prepared a checklist of the main elements of the disaster plan, which included 72 questions rated on a 3-point Likert scale, with the maximum and minimum scores being 140 and 0, respectively. The checklist was based on 13 functions, and an expert team performed the evaluation [15]. In this study, trained standardized patients (SPs) were involved in evaluating triage accuracy, time spent for patient assessments, and until the patients received definitive care. This is considered a crucial strategy in assessing hospital team performance [17].

Standardized patients (SPs) and evaluators assessed the response team’s performance. The evaluators assessed the major treatment accuracy, triage, and treatment areas. The SPs evaluated the accuracy of triage and the time taken for patients to receive treatment. They also documented the time they spent in the ED before being sent to the OR, ICU, and ward as an outcome measure [17]. Applying a simulation strategy with SPs improves accuracy, allows learners to manage anxiety, and develops critical thinking to enhance healthcare provider performance [17]. These well-trained individuals evaluate the players (trainees) performance during the final debriefing. Additionally, trained SPs can assess the skills acquired by the players, which helps evaluators accurately gauge the players skill development ^(17^Because SPs were recruited, no additional observers were needed [17].

### Drill performance – patient treatment, tracking and disposition /discharge

Disaster patient tracking begins with identifying and registering injured individuals, assigning them unique numbers, and recording their information, including medical conditions, from triage tags. Their treatment area in the ED is determined, and they are then tracked as they are either sent for further investigations or disposed to the wards, ICU, OR, morgue, transfer, or discharge. Tracking disaster patients is essential for successfully coordinating disaster responses. Timely, accurate, and accessible information about patients, their injuries and conditions, the services rendered at the scene, and their final discharging states is also necessary [48].

In this study, the triage category for SPs was accurate; for most cases (67%), the ED team took less than five minutes to examine the patient while 95% of patients were transferred to definitive care, including the ICU, OR, and wards, in less than 2.5 h. Approximately 65% of the patients in the drill required other treatment, including movement to other treatment zones, which is consistent with a previous study [49]. However, there was a delay in performing circulation treatment due to the inability to track patients in the yellow area.

Poor communication in some areas hampered patient tracking and coordination in this study. This was primarily due to problems with communication tools, updates, and debriefing for the response team. This resulted in two patients’ worsening. Effective communication is essential for coordinating response activities to minimize secondary morbidity and disease [50]. The use of personal mobile phones meant that some respondents did not receive the initial incident notification from the hospital system, resulting in incomplete reception of important information and delayed updates about the situation. The battery problems with the hospital radios in some sections also hindered effective communication and highlight the importance of system maintenance for disaster risk management.

Briefing and debriefing sessions are crucial to ensure the flow of information in the event of communication failure or limitations during a disaster [51]. Briefings ensure that all healthcare providers in relevant departments know the goals, action plans, safety concerns, roles and responsibilities, and reporting structure. Conversely, debriefing is a feedback process that provides information on the event’s progress, along with the Incident Action Plan (IAP), new information, and potential hazards [51]. There was a breakdown in information flow at the triage station and other treatment areas to varying degrees and there was limited briefing and debriefing occurring. Developing a strategy for monitoring injured patients within the hospital and sharing the relevant information during disasters can help prevent interruptions in information flow. Based on these findings, it is crucial to prioritize improving disaster training programs and strategies including briefing and debriefing policies.

### Usefulness of the drill for improving disaster risk management

The feedback from all participants regarding the drill was overwhelmingly positive, with 87% strongly agreeing that the drill was realistic and all respondents reporting that the drill was appropriate for their respective roles. The players (medical staff) reported the highest scores in relation to the scenario realism and its usefulness in their roles. This is crucial because they acknowledge how well the simulations mimic and prepare them for real-life situations, including the victims’ dynamic clinical conditions and the severity of injuries. The medical staff can further improve their cognitive and motor skills by simulating real-world scenarios and responding appropriately to their roles as outlined in the hospital disaster manual [52, 53].

The pre-drill orientation session was considered part of the drill. Any introductory lecture will contribute to learning by addressing the importance and relevance of the issues of participants to attend to during the drill. The participants in this study strongly agreed with the statement “briefing and presentation made it simpler for me to understand and participate in the situation.” The intensive and flexible preparedness program, including lectures and posters, produced clear improvement for participants. The value of a drill on its own without pre-drill preparation has limited improvement in physicians’ knowledge. Pre-drill preparation is an educational opportunity that can improve outcomes and preparedness [54–57].

### Usefulness of the SPs and MAC-SIM in the drill

Participants’ feedback on using MAC-SIM cards during this drill was generally positive, with around 87% finding them useful and accurate. The accuracy of MAC-SIM cards has been evaluated as high as 96% during simulation exercises by 123 participants in other studies [18]. These results suggest that MAC-SIM cards should be used in functional drills of this nature across the Saudi health system.

Most instructors agreed that the SPs understood the drill’s objectives and aims and knew their roles and how to behave to provide a realistic scenario for the participants. While most instructors also reported that the SPs knew how to adjust the physiological parameters based on the players’ responses, they suggested that additional focus and training are required during the pre-drill period under the supervision of instructors.

The comparison of performance between experienced (paramedics) and inexperienced (nurses) (SPs) by instructors showed that paramedics with previous disaster knowledge, including participation in previous drills, outperformed nurses. This result contrasts with another study, which showed no significant difference between SPs of different occupations. In that study, fidelity (i.e., SPs followed their scripts) and participants’ performance were the same, regardless of their profession [20]. Volunteers who attended a disaster awareness course or participated in a previous drill may be more likely to be selected as SPs and can easily follow instructions during pre-drill training processes. Therefore, they could help provide a comprehensive, realistic scenario during the drill. Based on these findings, the most critical area for improvement is for volunteer SPs to develop their skills in adapting physiological parameters based on player responses. This could involve additional training in simulation techniques, feedback about tracking player responses, or more precise guidelines on how to adjust during the drill.

## Evaluation of the hospital surge capacity plan

The hospital surge capacity plan was generally successful, with only three main issues identified. The first concern is the need for qualified staff to be assigned to the ED. Previous research has shown that the number of resuscitation teams that can be established and working in parallel in the ED, particularly in the red zone, including the trauma bay, is a limiting factor in hospital capacity [58]. One hour after the hospital disaster plan was activated, only five physicians and seven nurses from other departments participated in the red and yellow areas, leading to the health deterioration of two patients. Second, some staff members were observed to be disengaged and unaware of their roles and responsibilities, particularly in the wards, potentially due to a lack of relevant skill or a general lack of interest in the drill or other priorities. Similar behaviors have been reported in other studies [55].

The last issue was the excessive use of resources in the ED. This can be addressed by increasing staff awareness regarding the use of resources based on the patient triage category and the activated hospital emergency level. It was emphasized that orientation sessions in pre-drill preparation are a significant learning opportunity to improve performance and readiness [58]. Testing a single function will not reveal the surge capacity because all hospital functions interact and can be limiting factors at different points during the response [58].

A well-trained and well-organized department may operate at a greater capacity than a department with more resources but insufficient plans and untrained staff. To define hospital capacity, conducting regular practical tests during office and non-office hours is crucial. This approach ensures preparedness and efficient resource use [19, 57, 58].

### Limitations

In this study, several factors were found to limit the performance of the hospital staff. Firstly, there were time gaps between drills due to COVID-19, which meant that the hospital staff needed to be more aware of their roles, responsibilities, and the details of the hospital disaster risk management plan. Secondly, the drill only involved the hospital and did not include prehospital response or other external response agents, such as Emergency Medical Services, Red Crescent, and civil defense. Third, the standardized patients (SPs) were only used in the ED, which could affect the realism of the simulation for staff in other departments.

The authors recommend using MAC-SIM cards with SPs in various hospital units such as wards, ICU, and OR to enhance the realism of drills and effectively assess the hospital’s overall capacity [58]. This assessment should cover all aspects of the disaster rescue chain, from the initial evaluation at the disaster site to command and control, search and rescue, field care, and the transfer of victims to hospitals.

Furthermore, participants’ low feedback response rate (16%) after the drill was primarily due to the medical staff’s limited awareness about its importance in enhancing the hospital’s response to disasters, coupled with their busy daily duties. In future exercises, the significance of post-drill feedback should be reinforced to all participants, and adequate time allocated for them to provide their input.

## Conclusion

This study is the first in Saudi Arabia and the Gulf region to use simulated patients (SPs) with MAC-SIM cards in functional drills for disaster risk management and response. Despite limited resources and capabilities, the hospital team’s knowledge and performance, including decision-making, triage accuracy, coordination and communication, and surge capacity plan management, can be improved through these simulation exercises. Pre-drill preparation provides a valuable learning opportunity to enhance the performance and readiness of participants. Employing multiple evaluation techniques from different perspectives can help identify participants’ weaknesses and strengths, highlighting areas in hospital disaster plans that require further improvement. Limited and poorly designed disaster health drills can impact disaster response and reduce disaster awareness among front-line healthcare providers. Frequent competency-based drills, e.g., twice a year, are fundamental for improving staff performance during disaster response and maintaining the healthcare system’s resilience to disasters. Future research is required to assess the performances of healthcare providers in all parts of the rescue chain while using SPs with MAC-SIM cards. These data could be a valuable resource in other Arabic and middle-income countries where disaster medicine is still developing.

## Electronic supplementary material

Below is the link to the electronic supplementary material.

Supplementary Material 1

Supplementary Material 2

Supplementary Material 3

## Data Availability

The datasets generated during and/or analyzed during the current study are available from the corresponding author upon reasonable request.
