# Supplementary material for: Assessment of the effectiveness of hospital external disaster functional drills on health care receivers’ performance, using standardized patients and mass cards simulation: a pilot study from Saudi Arabia
Source: BMC Emerg Med. 2024 Sep 27;24:175. doi: 10.1186/s12873-024-01095-7 (PMC11438112; doi:10.1186/s12873-024-01095-7)
Supplement: Supplementary file 1 — Supplementary Material 1 [file 12873_2024_1095_MOESM1_ESM.pdf]

## Actor Exercise Assessment Form

Patient card NO (    )
Please complete the following questions at the conclusion of the exercise & be accurate with your answers. Your cooperation is important and is appreciated.

**Field Assessment and Treatment:**

**1. Initial Contact, Triage, and lifesaving procedures**

a. How long did it take for the first responder (EMS) to contact you? ________

b. When was the first triage done? ------------

c. How long is a primary triage taken from the first responder?

☐ < one mint ☐ > one mint ☐ one mint

d. Where have you examined on the scene more than once? ☐ Yes ☐ No

e. Whom did you talk to, or whom were you assessed by (list all)?

☐ Fire ☐ EMS ☐ Police ☐ Other ________________

f. If you received a multicolored triage tag, what was the BOTTOM color when it was first given to you?

☐ Green ☐ Yellow ☐ Red ☐ Black ☐ Never received a tag

2. Did the first responder do the lifesaving procedures according to your case?

☐ Yes ☐ no

4. Did lifesaving procedures done in proper time that can prevent the death /complications? (According to your case in storyboard)

☐ yes ☐ no

**Basic Treatment:**

1. If conscious, did someone explain your treatment? ☐ Yes ☐ No
2. If conscious, were you given clear instructions? ☐ Yes ☐ No
3. What treatment was given?

_______________________________________________________________

_______________________________________________________________

_______________________________________________________________

**Transportation**

1.Did secondary triage be done before transpiration**?**

2. What is your category before transportation?

3.What is the EMS Approach in the scene?

☐ Scope &run ☐ stay and play

**Hospital**

1. Which hospital did you go to (for local and national drill)?
   ______________________________
2. Did you categorize by the triage team at the hospital triage station?
   ☐ Yes ☐ no
3. What is the color tag given to you?
   ☐ Green ☐ Yellow ☐ Red ☐ Black
4. Once at the hospital, how long was it until someone examined you?
   ☐ Less than 5 minutes ☐ 5 minutes ☐ 10 minutes ☐ 15 minutes ☐ Over 15 minutes ☐ I was never examined at the hospital
5. What was the emergency department time spent in the emergency department before you send to OR /ICU /Ward?
   a. Less than 2.5 h
   b. 2.5 h
   c. More than 2.5 h

**Emergency department time (EDT) was defined as the time from ED triage that is necessary for adequate diagnosis, treatment, and actual disposition of critically and moderately injured patients.*

**Exercise Design:** Did you observe any problems during your participation in the exercise? What improvements would you suggest?

______________________________________________________

______________________________________________________

______________________________________________________

**DO NOT LOSE THIS CARD & Thank you for your participation**
