# Supplementary material for: Assessment of the effectiveness of hospital external disaster functional drills on health care receivers’ performance, using standardized patients and mass cards simulation: a pilot study from Saudi Arabia
Source: BMC Emerg Med. 2024 Sep 27;24:175. doi: 10.1186/s12873-024-01095-7 (PMC11438112; doi:10.1186/s12873-024-01095-7)
Supplement: Supplementary file 3 — Supplementary Material 3 [file 12873_2024_1095_MOESM3_ESM.pdf]

Field And IN Hospital Treatment List:

**Airway RX**	**YES /Time**	**No**		**Breathing RX**	**YES /Time**	**No**
Naso / orophar. tube (1min)				Oxygen (2min)		
Laryng. Mask (3min)				Vent -support (req. 1 staff)		
Trach. intub.(7min/2min)				Needle thorac.(4min)		
Cricothyroidotomy (5min)				Chest drain (7min)		
Gastric tube (3min)						
**Circulation, fluid, blood RX**	**YES /Time**	**No**		**Fracture -stabilization, bandage**		
Iv. needle (3min)				Covering bandage (4min)		
Analgesia (Needle+2min)				Neck Stabilis.(5min)		
Urinary cath.(4min)				Pelvic girdle /wrap (4min)		
Diagn . perit. lav (5min)				Splinting of limb (7min)		
IV.fluid (needle +2min)						
Intraoss needle (3min)						
Tourniquet (1 min)						
Blood						
**Examination: CT, USG,XRAY**				**Planned Surgery**		
USG (FAST) (5min)				Craniotomy		
				Vascular repair		
				Angiogr./embolis		
CT (45/15MIN)				Maxillofac. repair		
				Wound debridm		
Lung X ray (5min)				Eye repair		
				Fasciotomy		
Skel. XRAY (8min)				Neck exploration		
				External fixation		
				Amputation		
				Thoracotomy		
				Laparotomy		
				Pelvic pack		
